# Phosphorylation of Calpastatin Negatively Regulates the Activity of Calpain

**DOI:** 10.3390/life13030854

**Published:** 2023-03-22

**Authors:** Yuqiang Bai, Chengli Hou, Caiyan Huang, Fei Fang, Yu Dong, Xin Li, Dequan Zhang

**Affiliations:** Institute of Food Science and Technology, Chinese Academy of Agricultural Sciences/Key Laboratory of Agro-Products Quality & Safety in Harvest, Storage, Transportation, Management and Control, Ministry of Agriculture and Rural Affairs, Beijing 100193, China

**Keywords:** tenderization, structural stability, serine

## Abstract

Tenderness is an important characteristic of meat quality. Calpastatin and calpain play important roles in meat tenderization. However, it is not clear how phosphorylation affects the regulation of calpastatin on μ-calpain and, consequently, meat tenderness. Calpastatin with high and low phosphorylation levels were obtained in vitro corresponding to the treatments by protein kinase A (PKA) and alkaline phosphatase. Then, calpain was incubated with calpastatin with different phosphorylation levels, and the effect of calpastatin on calpain activity under different phosphorylation levels was analyzed. The results showed that PKA promoted the phosphorylation of calpastatin, and a high phosphorylation level was maintained during incubation. The degradation rate of μ-calpain in AP group was higher than that in the other groups, meaning there was lower inhibition of calpastatin on calpain activity. The degradation of calpastatin was lower and its structure was more stable after phosphorylation. One more serine 133 site of calpastatin was identified in PKA group compared with the other groups. Phosphorylation at serine 133 of calpastatin enhanced its inhibition on calpain activity by maintaining its structural stability, thus inhibiting the tenderization of meat.

## 1. Introduction

Tenderness is the primary consideration for consumers when purchasing meat products [1]. Factors related to meat tenderization include proteolysis of muscle myofibrillar fibers, collagen content, sarcomere length, intramuscular fat, and protein denaturation [2]. The final tenderness of meat depends on the changes of structural proteins in muscle [3]. The calpain system is a calcium-dependent endogenous protease system responsible for proteolysis of myofibrillar proteins in muscle fibers, resulting in tenderization [4]. Differential expression of members in calpain system regulates meat tenderness, especially µ-calpain and calpastatin, which may be considered as biomarkers of tenderness [5]. μ-calpain is an endogenous enzyme responsible for tenderizing meat postmortem. In the presence of calcium, the complete 80 kDa μ-calpain catalytic subunit autolyzes into intermediates (78 kDa), eventually forming active subunits (76 kDa) [6]. μ-calpain was activated by Ca^2+^ during three days postmortem to autolysis and exert its hydrolytic activity. Autolysis of calpain reduces the concentration of Ca^2+^ required to activate calpain. Autolysis of calpain is associated with activation of calpain and degradation of proteins at early postmortem. Calpain damages the ultrastructure of muscle by degrading proteins in muscle fibers and promotes fragmentation of myofibrillar fibers and tenderization of meat [7]. The earlier the autolysis state of calpain occurs, the earlier degradation of myofibrillar proteins such as titin and desmin [8]. The degree of calpain activation was positively correlated with muscle tenderness [4]. Calpain in tender meat has a higher degree of degradation, a faster activation speed, and a greater effect on muscle tenderization [9].

Calpastatin is an endogenous inhibitor of calpain that regulates the degradation of myofibrillar protein in vivo. Calpastatin regulates protein conversion, muscle differentiation, and growth in living animals [10]. The gene of calpastatin is closely related to the tenderness of livestock meat [11]. Calpastatin regulates the degradation of muofibrillar protein in postmortem muscle tissue and the rate and degree of postmortem meat tenderization [3]. Calpastatin specifically inhibits the activity of calpain. The higher the activity of calpastatin, the less tender the meat. When calpain is activated by Ca^2+^, calpastatin rapidly binds to the activated calpain and inhibits its activity [12]. The content of calpastatin in muscle is gradually decreased after slaughtering, which is caused by degradation by proteolytic enzymes such as calpain, cathepsin, and proteasome. The activity of calpastatin is closely related to its expression, and the activity of calpastatin decreased due to its reduced content [13]. The inhibitory activity of calpastatin was positively correlated with the shear force of meat [14]. The degradation rate of calpastatin in meat with high tenderness was fast, and the inhibition ability on calpain activity was the lowest [15]. The degradation rate and deactivation of calpastatin are related to protein hydrolysis and meat tenderness. The degradation rate and deactivation of calpastatin are involved in the regulation of meat tenderness through the interaction with calpain [16].

Protein phosphorylation, a common post-translational modification of proteins, is involved in regulating many important physiological processes [17] and plays an important role in the formation of meat quality. Protein phosphorylation is negatively correlated with meat tenderness, and many phosphorylated proteins have been identified in meat related with tenderness [18]. Both calpain and calpastatin are phosphorylated proteins. There are 9 and 8 phosphorylation sites on the 80 kDa large subunit of μ-calpain and m-calpain, respectively [19]. The activity of calpain in tumor cells increased after phosphorylation and the regulation of cell cycle was enhanced, thus promoting the adhesion, migration, and diffusion of tumor cells [20]. Phosphorylation of μ-calpain by protein kinase enhances its hydrolytic activity [21]. Both dephosphorylation and phosphorylation catalyzed by AP and PKA positively regulate the activity of μ-calpain. The phosphorylation of Serine255, 256, and 476 at μ-calpain catalyzed by PKA may improve its activity by changing its hydrolytic ability and activity regulation ability [22]. As early as 2011, a cDNA library of goat calpastatin was constructed and the phosphorylation sites of amino acid residues were predicted. The sites included 42 serines, 18 threonines, and 1 tyrosine sites. The study also identified five specific sites where protein kinase C catalyzed the phosphorylation of calpastatin. Amino acid residues corresponding to the haplotype closely related to tenderness in the calpastatin gene can be phosphorylated by protein kinase [23]. Phosphorylated calpastatin affected its inhibitory efficiency in cells [24]. Phosphorylation of calpastatin was involved in regulating the stability of the calpain-calpastatin system [25]. Phosphorylated calpain catalyzed by PKA was more sensitive to the inhibition of calpastatin, and calpastatin had a stronger inhibition on its activity [26]. These findings suggest that phosphorylation of calpain and calpastatin is involved in meat tenderness formation.

However, how the phosphorylated calpastatin exerts its inhibitory effect on calpain remains unclear. A hypothesis is that phosphorylation changes the inhibition of calpastatin on calpain activity by changing the structure of calpastatin, which may be due to the binding of phosphate group on calpastatin varying the affinity of calpastatin and calpain. Therefore, models of calpastatin with different phosphorylation levels in vitro were constructed to explore the effects of phosphorylated calpastatin on the activity of calpain in order to understand the mechanism of meat tenderness from a new point of view.

## 2. Materials and Methods

### 2.1. Reagents

μ-calpain (208712) and calpastatin (208900) were obtained from Calbiochem (San Diego, CA, USA) and Merck-Millipore (Burlington, MA, USA), respectively. Protein kinase A (P2645), alkaline phosphatase (P0114), ATP (A7699), and primary antibody against calpastatin (C270, 1:500 dilution) were obtained from Sigma-Aldrich (St. Louis, MO, USA). Primary antibody against calpain (MA3-940, 1:1000 dilution), Pro-Q Diamond (MPP33300), and SYPRO Ruby (S12000) were obtained from Thermo Fisher Scientific (Waltham, MA, USA).

### 2.2. Experimental Design

**Figure 1:**
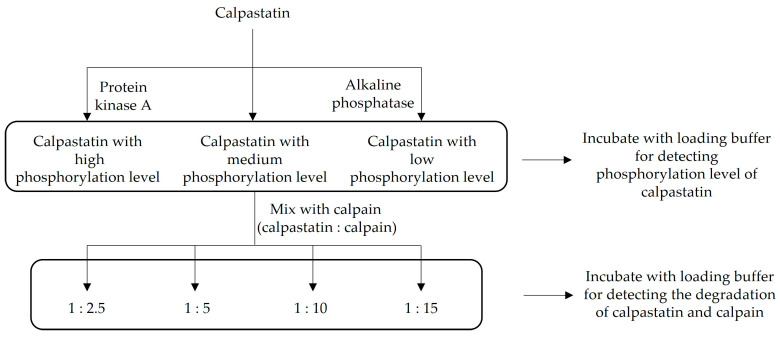
Flow chart of experimental design.

Equal amounts of calpastatin (200 μg) were incubated with kinase or phosphatase to regulate its phosphorylation levels as follows: (1) adding protein kinase A (PKA) as a high phosphorylation level group; (2) adding alkaline phosphatase (AP) as a low phosphorylation level group; (3) control group (control) without addition of PKA or AP (Figure 1). The contents of PKA and AP were 10 and 20 U per 100 μg protein, respectively. The added content of ATP was 2 μM per 100 μg protein. The final volume of each group was 2 mL and was adjusted with incubating buffer (50 mM Tris, 10 mM DTT and 10 mM MgCl_2_, pH 6.8). All groups were incubated at 30 °C for 30 min. Part of each group (50 μL) was mixed with the same volume of loading buffer and incubated at 100 °C for 5 min.

The other part of treated calpastatin (800 μL) with different phosphorylation levels was divided into 4 groups, mixed with pure μ-calpain at the ratio of 1:2.5, 5, 10 and 15, respectively. CaCl_2_ solution was added to all groups to adjust the final concentration of Ca^2+^ in the system to 0.05 mM and the final volume to 1125 μL. Samples were collected at 4 °C for 1, 2, 12, and 24 h during incubation.

### 2.3. Phosphorylation Levels of Calpastatin

Samples (15 μg) were loaded onto polyacrylamide gels (10% separation gel and 4% stacking, corresponding voltages were 110 and 70 V). After electrophoresis, gels were stained with Pro-Q Diamond and SYPRO Ruby for phosphorylated and total calpastatin, respectively. The ChemiDoc^TM^ MP Imaging System (Bio-Rad, Hercules, CA, USA) was applied for scanning, and Quantity One software (version 4.62, Bio-Rad, Hercules, CA, USA) was used to quantify and evaluate the phosphorylation levels of calpastatin.

### 2.4. Degradation of μ-Calpain and Calpastatin

SDS-PAGE electrophoresis and western blotting were used to determine the degradation of μ-calpain and calpastatin. The polyacrylamide gels for calpain comprised 8% separation gel and loading volume was 30 μg. After electrophoresis, the bands were transferred to the PVDF membrane and performed at 100 V for 100 min. After western blotting, PVDF membrane was incubated with antibodies of μ-calpain and calpastatin, respectively. The degradation rate of the 80 kDa large subunit of μ-calpain was used to represent the activity of μ-calpain.

### 2.5. Identification of Protein Sites by Liquid Chromatography-Tandem Mass Spectrometry (LC-MS/MS)

The proteins for LC-MS/MS identification should undergo electrophoresis, Coomassie brilliant blue staining and cleaning with ultrapure water and gel digestion bleaching solution (50% acetonitrile, 25 mM NH_4_HCO_3_). Cycles of dehydration with acetonitrile and cleaning with NH_4_HCO_3_ fellow were performed. It was then incubated and digested on ice and 37 °C, respectively. A proportion of 0.1% trifluoroacetic acid was added to stop the reaction after digestion overnight. Finally, LC-MS/MS (Thermo Q-Exactive, Thermo Scientific, Waltham, MA, USA) was used to detect and Mascot 2.2 search engine was used to identify proteins.

### 2.6. Statistical Analysis

The data were analyzed using SPSS Statistic 21.0 (IBM, Armonk, NY, USA). Generalize linear model (GLM) was used to analyze the variations of phosphorylation level of calpastatin and the degradation of μ-calpain. The differences among the means were compared using the Fisher’s Protected Least Significant Difference (LSD) test (*p* < 0.05). All results were presented as means and standard deviations.

## 3. Results

### 3.1. Phosphorylation Level of Calpastatin

**Figure 2:**
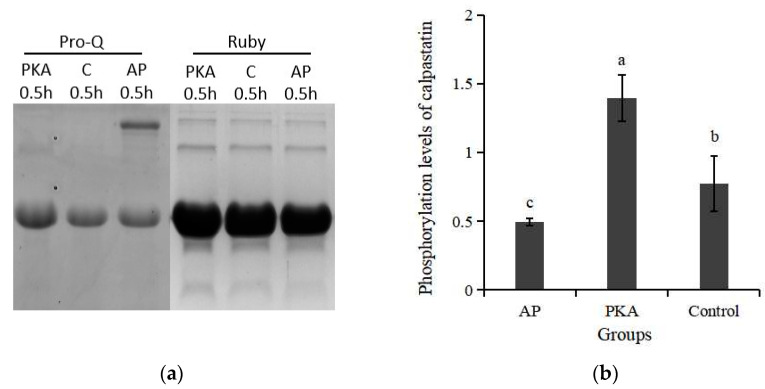
Phosphorylation images of calpastatin in the three groups after incubation at 30 °C for 30 min. (**a**) Phosphorylation of calpastatin in the three groups after incubation at 30 °C for 30 min; (**b**) relative phosphorylation levels of calpastatin in the three groups. a–c represents significant difference between groups (*p* < 0.05).

In order to determine the effect of phosphorylated calpastatin on the activity of μ-calpain, calpastatin with different levels of phosphorylation were obtained by adding PKA and AP. The phosphorylation level of calpastatin after phosphorylation and dephosphorylation treatment is shown in Figure 2. The phsosphorylation level of calpastatin in PKA group was significantly higher than that of the other groups (*p* < 0.05). The phosphorylation level of calpastatin in AP group was significantly lower than that in the control group. These results indicated that AP and PKA catalyzed dephosphorylation and phosphorylation of calpastatin, and calpastatin with different phosphorylation levels were obtained.

**Figure 3:**
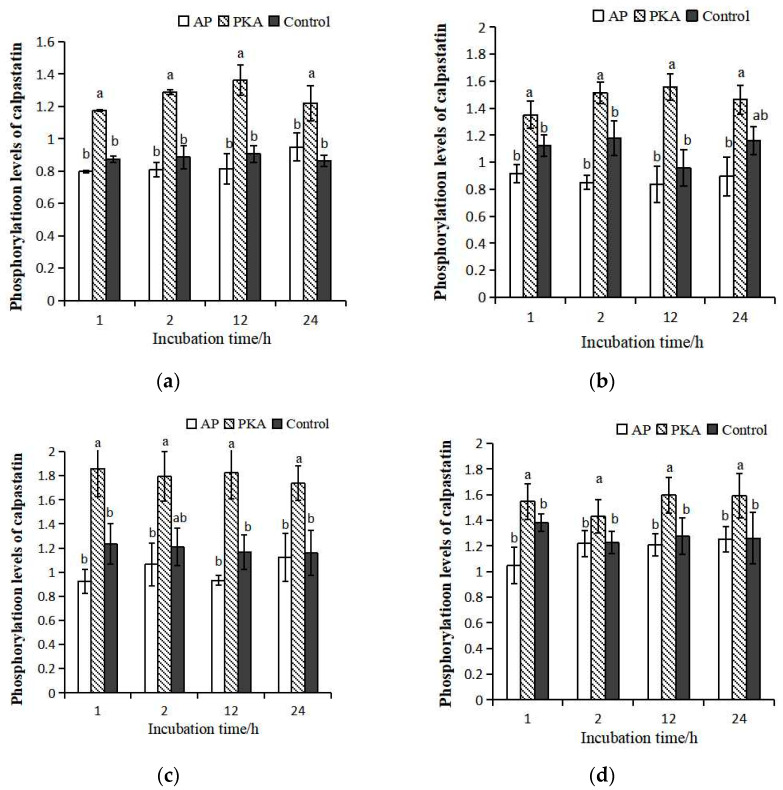
Phosphorylation levels of calpastatin in three groups with different ratios between calpastatin and calpain during incubation at 4 °C. Calpastatin:calpain = 1:2.5 (**a**); 1:5 (**b**); 1:10 (**c**); 1:15 (**d**). a–b represents significant difference between groups (*p* < 0.05).

Calpastatin with different phosphorylation levels were mixed with μ-calpain at ratios of 1:2.5, 1:5, 1:10 and 1:15. The phosphorylation levels of calpastatin in each system were detected at 1, 2, 12, and 24 h during incubation (Figure 3). In general, the changes of phosphorylation level in the four incubation systems among the three groups were consistent as the extension of incubation time. In the incubation system with different ratios of calpain and calpastatin, there was no significant difference between the control group and the AP group. In the system of calpastatin:calpain = 1:2.5 and 15, the phosphorylation level of calpastatin was significantly higher in the PKA group than that in the other groups. There was no significant difference in the phosphorylation level of calpastatin between the PKA group and control group at 24 h incubation in the system with calpastatin:calpain = 1:5, but it was significantly higher than that in control group at other time points. In the system of calpastatin:calpain = 1:10, the phosphorylation level of calpastatin in PKA group was not significantly different from that in control group at 2 h of incubation but was significantly higher than that in control group and AP group at other time points. In the system of calpastatin:calpain = 1:15, the phosphorylation level of calpastatin was the highest in the PKA group. The results indicated that the effects of PKA on the phosphorylation of calpastatin were well maintained during incubation.

### 3.2. Degradation of μ-Calpain

**Figure 4:**
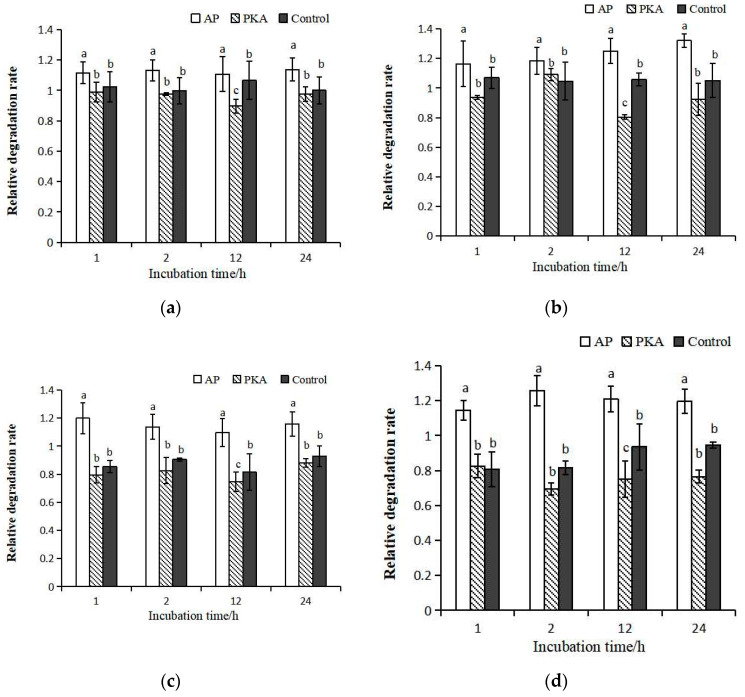
Relative degradation rate of μ-calpain in three groups with different ratios between calpastatin and calpain during incubation at 4 °C. Calpastatin:calpain = 1:2.5 (**a**); 1:5 (**b**); 1:10 (**c**); 1:15 (**d**). a–c represents significant difference between groups (*p* < 0.05).

The degradation of the large subunits represents μ-calpain activity (Figure 4). The degradation degree of μ-calpain with different ratios between calpastatin and calpain was consistent during incubation at 4 °C. The highest degradation degree was found in the AP group during incubation. The degradation degree in PKA group was significantly lower than that in control group at 12 h of incubation. There was no significant difference in degradation degree between the PKA group and control group at other time points. The different ratios between calpastatin and calpain had no significant effect on the degradation of calpain.

### 3.3. Degradation of Calpastatin

**Figure 5:**
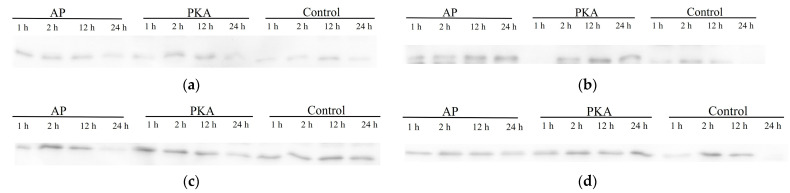
Degradation of calpastatin in three groups with different ratios between calpastatin and calpain during incubation at 4 °C. Calpastatin:calpain = 1:2.5 (**a**); 1:5 (**b**); 1:10 (**c**); 1:15 (**d**).

There was obvious degradation of calpastatin in the calpastatin:μ-calpain = 1:2.5 incubation system, and the degradation of calpastatin was significantly lower in PKA group than that in other treatment groups (Figure 5). The degradation degree of calpastatin in PKA group was significantly lower than that in AP group when calpastatin:μ-calpain was 1:5 and 1:10, respectively. The degradation degree of calpastatin in the control group was higher than that in the AP group within incubation in the calpastatin:μ-calpain = 1:5 incubation system. The degradation degree of calpastatin in the control group was lower than that in the AP group within incubation in the calpastatin:μ-calpain = 1:10 incubation system. In the calpastatin:μ-calpain 1:15 incubation system, the degradation degree of calpastatin was the highest in the control group. In the control group, bands of calpastatin could hardly be seen after incubation.

### 3.4. Phosphorylation Sites of Calpastatin

Two phosphorylation sites of calpastatin were identified in the PKA group (Table 1): Threonine 135 (Thr 135) and Serine 133 (Seer 133), respectively. Both sites are on the same phosphorylated peptides and are separated by only one amino acid. Only one phosphorylation site, Threonine 135, was identified in the control group and the AP group. There was one more phosphorylation site on serine in PKA than in the other two groups.

## 4. Discussion

After incubating at 30 °C for 30 min, calpastatin with high, medium, and low phosphorylation levels were obtained, corresponding to PKA, control and AP groups. It indicated that calpastatin with different phosphorylation levels were successfully constructed in this study, and PKA and AP could effectively catalyze the phosphorylation and dephosphorylation of calpastatin. After mixing with different ratios of calpain and incubating at 4 °C, the high phosphorylation level of calpastatin in the PKA group was maintained. There was no significant difference between the AP group and the control group in the phosphorylation level of calpastatin, which might be due to the decrease of the phosphorylation level of calpastatin in the control group or the increase of the phosphorylation level of calpastatin in the AP group. The amount of ATP in this study could only catalyze phosphorylation of up to 200 μg protein, and the amounts of PKA and AP in different groups were added according to the standard of 200 μg protein. Therefore, the phosphorylation level of calpastatin should be effectively inhibited by AP in the AP group, and the phosphorylation level of calpastatin would not increase. The reason why there was no significant difference in the phosphorylation level of calpastatin between the control group and AP group was that the phosphorylation level of calpastatin in the control group decreased.

Calpain performs its proteolytic activity through autolysis degradation, so the degradation degree of μ-calpain is often used to indicate its activity. The faster the calpain degradation rate, the faster the activation rate, and the higher the calpain activity [27]. In the process of incubation with calpastatin, the activity of calpain in the PKA group was significantly lower than that in the AP group. However, in incubation models with different ratios of calpastatin and calpain, the changes of calpain activity were consistent among different groups in this study. It indicated that the ratio of calpastatin and calpain did not affect calpain activity during incubation in vitro. The possible reason for this result was due to the low incubation temperature of calpastatin and calpain or the relatively low content of calpastatin added in the system. Therefore, no regulation effect of phosphorylation on the inhibitory ability of calpastatin was detected except for 12 h of incubation. The results showed that the dephosphorylation of calpastatin in AP group reduced its inhibitory activity, leading to the increase of calpain activity [26]. Previous studies showed that compared with dephosphorylated calpastatin, phosphorylated calpastatin catalyzed by PKA has a stronger inhibition ability on m-calpain activity and phosphorylation was involved in regulating the inhibition ability of calpastatin [9]. Previous studies also confirmed that reversible post translational modifications of calpastatin can change its ability to recognize calpain and significantly reduce its inhibitory efficiency [28]. In rat brains, phosphorylated calpastatin showed significantly reduced inhibitory efficiency, especially for m-calpain [4]. In skeletal muscle, the same post translational modification changes the specificity of the calpastatin. Calpastatin inhibits calpain activity by directly binding with calpain [13]. The different effects of the same modification on the activity of calpastatin and calpain may be due to the differences of species and system. The characteristics of calpastatin were controlled by the coordination of PKA and AP [29]. Protein kinase A promoted the formation of calpastatin aggregation and reduced the amount of direct interaction with calpain. Alkaline phosphatase promoted the release of calpastatin from aggregation, thereby inhibiting the activity of calpain [30]. Phosphorylation of calpastatin caused it to accumulate and sequester near the nucleus, whereas dephosphorylated calpastatin was free in the cytoplasm and exerted inhibitory activity [29]. In a study about the phosphorylation of calpastatin in human hematopoietic cell lines, more than 30% of 32P-labeled calpastatins with different molecular weights were found to be distributed in the membrane portion. Therefore, phosphorylation of calpastatin may participate in the regulation of the calpain-calpastain system through its subcellular distribution [25]. The formation of the calpain-calpastatin complex only occurred in the presence of relatively high Ca^2+^, which induced conformational changes and revealed the catalytic site [25]. This reversible process was followed by the removal of calpain’s N-terminal peptide domain, a process that further increases calpastatin’s affinity for the calpain [31]. The addition of phosphate groups determined the increase in Ca^2+^ concentration required to induce the formation of the calpain-calpastatin complex, which lead to a significant decrease in the inhibitory efficiency of calpastatin. Therefore, the phosphorylation of calpastatin represents a mechanism that can balance the actual amount of active calpastatin with the level of calpain to be activated [32]. In this study, exogenous PKA and AP were not sufficient to catalyze the phosphorylation and dephosphorylation of calpain, so it was speculated that there was no significant difference in the phosphorylation levels of calpain among the three groups during incubation. The differences in the degradation degree of calpain and calpastatin by calpain were caused by different phosphorylation levels of calpastatin. The inhibitory ability of dephosphorylated calpastatin on calpain activity was smaller than that of phosphorylated calpastatin catalyzed by PKA, and phosphorylation may play a positive role in regulating the inhibitory ability of calpastatin on calpain activity [33].

In the incubation process, the structure of calpastatin was relatively stable in the PKA group, and obvious degradation was observed in the AP group. It was speculated that the phosphorylation of calpastatin exerted high inhibitory activity by maintaining its structural stability. Protein post translational modifications affected the specificity and inhibition of calpastatin [28]. The intracellular cAMP level played a fundamental role in modifications, as it induces the release of calpastatin from the cytosol by activating PKA [34]. Calpain also degraded calpastatin, leading to the breakdown of intracellular structures. In medicine, calpain regulates important cellular processes through selective and limited target cell cleavage after the increase of Ca^2+^ [24]. However, under conditions of sustained Ca^2+^ increase, calpain proteolysis becomes excessive and its targets undergo extensive degradation. The transformation from physiological function to pathological function of calpain is due to the insufficient regulation of calpain proteolysis by calpastatin [35]. Thus, in protein misfolding diseases or disorders characterized by abnormal Ca^2+^ regulation, strategies to limit the effects of calpain activation are primarily based on calpastatin [36]. Phosphorylation of calpastatin was required to prevent structural damage to cells that may result from uncontrolled calpain activation. The increase concentration of Ca^2+^ promoted the activity of calpain and also promoted the formation of calpain-calpastatin complex and inhibited the activity of calpain [37]. In rat L8 myoblasts, the calpain-calpastatin system and caspase are involved in the degradation of fusion-related proteins. The level of calpain did not change significantly during the differentiation of L8 myoblasts. In contrast, calpastatin was significantly reduced before myoblast fusion and reappeared after fusion [38]. The decrease in calpastatin is due to a transient increase in caspase activity during myoblast differentiation [38]. Temporary caspase-induced reduction of calpastatin allows Ca^2+^ to promote calpain activation and proteolysis, which is necessary for cell fusion. In general, protein phosphorylation can promote the inhibitory activity of calpastatin by changing the structure of calpastatin from the following two aspects: (1) Protein phosphorylation promoted the binding of calpastatin and calpain, which helped the inhibition domain of calpastatin to better recognize and bind to calpain, thus inhibiting its activity. (2) Protein phosphorylation exerted its inhibitory activity by reducing the degradation by calpain. The degradation of unphosphorylated calpastatin by calpain may also be one of the reasons for the decreased phosphorylation level of calpastatin in the control group.

**Figure 6:**
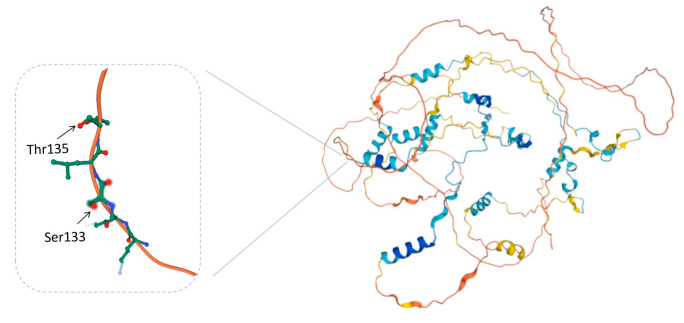
A 3D simulated diagram of calpastatin with two phosphorylation sits was obtained from the Uniprot database (http://www.uniprot.org/, accession number: P20810, accessed on 25 January 2023). The left side of the figure shows the specific sites in the calpastatin where calpastatin is phosphorylated.

The primary structure of calpastatin showed that there were four repeated calpain-inhibitory domains (I-IV). Each inhibitory domain contains three subdomains, and each subdomain can bind to one calpain molecule [39]. They have precise functions when interacting with amino acid sequences in the catalytic and regulatory subunits of calpain [40]. There are two extensions at the N-terminal of calpastatin peptides, namely XL and L domains. Phosphorylated calpastatin in these two domains has low inhibitory efficiency [41]. The L domain is involved in tight binding to the active site of calpain [42]. PKA can catalyze the phosphorylation of the L domain of calpastatin, thereby affecting the ability and specificity of calpastatin to inhibit calpain activity [24]. In the development of drugs, it has been found that the N-terminal regulatory domain of calpastatin undergoes selective splicing compared to the conserved and repetitive four inhibitory domains. Thus, a possible alternative strategy for regulating the calpain/calpastatin system may come from studying the effects of the non-inhibitory regions of the calpastatin molecule. The efficiency of calpastatin functions can be modulated by post translational modifications to non-inhibitory domains. The regulatory regions of calpastatin can be further diversified by alternative splicing within the L domain [35]. In this study, two phosphorylation sites were identified in the PKA group. Among these sites, Ser 133 was unique to this group. Therefore, it is speculated that the phosphorylation of calpastatin serine significantly enhances its phosphorylation level. The phosphorylation sites of 133 and 135 are located in the L domain, specifically at the corner of the peptide chain (Figure 6). Phosphorylation of these two sites may cause rotation, folding, dihedral angle, and rotation direction change of the peptide chain. This may further lead to changes in the spatial structure and intramolecular interactions of calpastatin, thereby altering the properties and functions of calpastatin. Therefore, phosphorylation may inhibit the activity of calpastatin by altering its spatial structure and inhibiting its binding to calpain.

Previous studies have shown that μ-calpain activity positively regulated meat tenderness and that both phosphorylation and dephosphorylation can promote calpain activity [21,26]. As an endogenous inhibitor of calpain, calpastatin significantly inhibited the activity of calpain [43]. The inhibitory activity of calpastatin was positively correlated with shear force, which was considered as a sign of shear force in aged beef [44]. Phosphorylation may alter the affinity of calpastatin to different calpain and ultimately alter proteolysis [45]. Protein phosphorylation also prevented the degradation by calpain by maintaining the structure of calpastatin, leading to a decrease in the content of calpastatin and thus reducing its inhibitory activity on calpain. In summary, both calpain and calpastatin are involved in the formation of meat tenderness, and protein phosphorylation plays an important role in the tender system of calpain. Protein phosphorylation tenderizes the muscle either directly by promoting the activity of calpain or by inhibiting the inhibitory activity of calpastatin to promote greater involvement of calpain in the formation of meat tenderness.

## 5. Conclusions

Protein kinase A significantly catalyzed the phosphorylation of calpastatin. The structure and phosphorylation level of phosphorylated calpastatin were well maintained during incubation with calpain. The phosphorylation calpastatin at serine 133 of promoted its inhibition on calpain activity. There may be two ways for phosphorylation to promote the inhibition of calpastatin on the activity of calpain. One is that phosphorylation exposes the domain on the calpastatin that plays an inhibitory role, promoting the binding of calpastatin and calpain and directly inhibiting its activity. The other is that phosphorylation keeps the structural stability of calpastatin, reduces the hydrolysis by calpain, and thus better displays its inhibitory activity. In general, phosphorylation of calpastatin negatively affects meat tenderization by inhibiting calpain activity. Inhibiting the phosphorylation level of calpastatin may be a new way to improve meat tenderness.

## Figures and Tables

**Table 1:** Identification of phosphorylated peptides and phosphorylated sites of calpastatin in the three groups with different phosphorylation levels.

	Phosphorylated Peptides	Motif	Phosphorylation Sites
PKA	KSLTPAVPVESKPDKPSGK	KEKKSLtPAVPVE	Thr 135
	SLTPAVPVESKPDKPSGK	KKKEKKsLTPAVP	Ser 133
Control	KSLTPAVPVESKPDKPSGK	KEKKSLtPAVPVE	Thr 135
AP	KSLTPAVPVESKPDKPSGK	KEKKSLtPAVPVE	Thr 135

## Data Availability

The data that support the findings of this study are available on request from the corresponding author.
